# Optimising the manufacture of perfluorocarbon nanodroplets through varying sonication parameters

**DOI:** 10.1016/j.ultsonch.2025.107332

**Published:** 2025-04-09

**Authors:** Christopher K. Campbell, Kirsten O’Brien, Dariusz Kosk, Robin M.H. Rumney, Peter Glynne-Jones, Peter R. Birkin, Gareth LuTheryn, Jeremy S. Webb, Eleanor Stride, Dario Carugo, Nicholas D. Evans

**Affiliations:** a Nuffield Department of Orthopaedics, Rheumatology and Musculoskeletal Sciences (NDORMS), University of Oxford, Oxford, UK; b Bone and Joint Research Group, Faculty of Medicine, University of Southampton, Southampton, UK; c School of Engineering, University of Southampton, Southampton, UK; d School of Pharmacy & Biomedical Sciences, University of Portsmouth, Portsmouth, UK; e Department of Chemistry, University of Southampton, Southampton, UK; f National Biofilms Innovation Centre (NBIC), School of Biological Sciences, University of Southampton, Southampton, UK; g Institute of Biomedical Engineering, University of Oxford, UK

**Keywords:** Particle manufacture, Perfluorocarbon nanodroplet, Ultrasound

## Abstract

Perfluorocarbon nanodroplets (PFC-NDs) are promising ultrasound-responsive theranostic agents with applications in both diagnostic imaging and drug delivery. The acoustic vaporisation threshold, extravasation potential, and stability of PFC-NDs are all affected by their size. However, methods to ensure reproducible size and concentration during production by sonication are lacking. To address this need, we examined the effect of temperature, sonication time, sonication intensity, PFC concentration and sonicator tip height on ND characteristics. PFC-NDs with a perfluoro-n-pentane (PFP) core and a phospholipid shell were manufactured by probe-sonication. Pulsed sonication was used to maintain the sample temperature below the boiling point of PFP. Median particle diameter was measured using nanoparticle tracking analysis. PFC-ND diameter increased with increasing PFP concentration, with a stronger relationship as sonicator tip height increased. Above 5% v/v PFP, there was a qualitative increase in the number of particles visible by light microscopy. Increasing the sonication duration did not yield a significant change in ND size. A minimum amplitude of 60% was required for mixing to occur, with amplitudes of 80% and 100% resulting in foam production. Sonicator power output was linear with respect to time but differed depending on sample volume, composition, and vessel geometry. This study indicates that controlling the processing parameters can facilitate reproducible manufacturing of PFC-NDs.

## Highlights

• A repeatable positioning system was developed to control the sonicator tip location.
• Increasing the volumetric concentration of PFP increases the median diameter of NDs.
• Increasing the sonication amplitude did not significantly change the ND median diameter.
• Pulsed sonication enables samples to be kept under 16 °C in a water-ice bath.

## 1 Introduction

Nanoemulsions have been investigated for decades as circulating agents for applications in magnetic resonance and ultrasound contrast imaging as well as targeted delivery of therapeutics [1], [2], [3]. A subset of these nanoemulsions comprises perfluorocarbon nanodroplets (PFC-NDs). These are sub-micrometre in diameter and consist of a spherical liquid perfluorocarbon core stabilised by an amphipathic surfactant monolayer, such as a phospholipid. PFC-NDs have previously been investigated as agents for drug and gas delivery as well as to enhance ultrasound contrast imaging. This is due to their ability to undergo vaporisation into gas bubbles upon ultrasound exposure [4], [5]. The subsequent collapse of the resulting microbubbles can be used to deliver therapeutic effects at a target site [6]. As PFC-ND diameter increases, the pressure required to vaporise them decreases [7]. PFC-NDs containing fluorine-19 have also been employed in magnetic resonance imaging (MRI) as a contrast agent. The main advantages of PFC-NDs over microbubbles as contrast agents are their longer circulatory half-life and ability to more effectively perfuse the microvasculature [8]. PFC-NDs have previously been studied as drug and gas delivery systems in applications including cancer therapy, blood substitution, and blood–brain barrier permeation [3], [9], [10], [11], [12]. Currently, one perfluorodecalin-core fluorocarbon ND is approved for use as a blood substitute in Russia, Kazakhstan and Mexico [13].

A common laboratory technique for creating NDs consisting of perfluorocarbons heavier than perfluorobutane is probe sonication. This process involves using low frequency (i.e., typically 20–60 kHz), high-intensity ultrasound to induce the formation of bubbles by cavitation, which, upon collapse, generate strong shear forces in the liquid. When applied to a mixture of two immiscible fluids, these forces result in the generation of droplets that break up into progressively smaller ones over time. This process is less costly than alternative techniques, such as high-pressure homogenisation, can create smaller droplets than current microfluidic-based techniques, produces fewer surfactant-only structures than gas condensation methods, and avoids harmful solvents used in current solvent exchange methods [21], [22], [23]. The main disadvantage of sonication is that it is a stochastic process, and thus, the ND size distribution can vary substantially from batch to batch [8]. There is, therefore, a need to tightly control the possible variables involved, so that the resulting ND size distribution is more predictable and reproducible. The specific sonication and formulation parameters used in the production of NDs are not consistently reported in published studies (Table 1). For example, the rationale for the choice of parameters such as sonication amplitude or PFC concentration is not always presented, while geometric parameters such as the relative location of the sonicator tip with respect to the liquid sample and the type or size of the container are often not specified. In addition, the reported sonication power values vary considerably. Typically, a sonicator has a known maximum power output, with sonication intensity modified by changes in the percentage amplitude setting.

**Table 1:** Selection of studies using sonication to manufacture PFP-core NDs.

Study	Sonicator Model	Power (W)	Duration (On Time)	Duty Cycle	PFP Volumetric Concentration*	Droplet Diameter	Sizing Method
Ferri et al. [8]	Model 120 Sonic Dismembrator, Fisher Scientific, UK	48 or 72 W	20 s, 40 s or 60 s	15% or 30%	5.00–15.00% v/v	200–300 nm	DLS
Zhang P & Porter T [14]	VC505, Sonic & Materials, USA	Unspecified	30 sec	Unspecified	3.00% v/v	193.3 nm	DLS
Matsuura et al. [15]	Sonicator 3000, Misonix, Bioventus Inc., USA	10 W	5 min	50%	∼2.90% v/v	100-300 nm	DLS
Williams et al. [16]	S-450D, Branson Ultrasonics, USA	2 W	5 min	50%	3.13% v/v	221 nm	DLS
Reznik et al. [17]	Sonifier 250, Branson, USA	Unspecified	40 s	80%	5.00% v/v	∼400 nm	DLS
Aliabouzar et al. [18]	Q500, Qsonica, USA	125 W	10 s	Unspecified	9.09% v/v	890 nm	RPS
Ho YJ & Yeh CK [19]	UTR200, Dr Hielscher Company, Germany	200 W	20 min	Unspecified	6.98% v/v	373 nm	NTA
Zhang et al. [20]	Unspecified	90 W	36 min to 75 min	Unspecified	1% v/v	110 nm	DLS

DLS= dynamic light scattering, RPS= resistive pulse sensing, NTA= nanoparticle tracking analysis, * volumetric concentration of PFP prior to ND manufacture by sonication

Many commercial sonicators may not report absolute power output or amplitude. Unless the sonicator has a real-time power readout, it may not be possible to report it accurately as the power output is adjusted to allow for a consistent displacement amplitude. Despite the range of sonication intensities and durations used to manufacture PFP-core NDs, the majority of reported particle diameters are within a range of 100–400 nm. Methods such as dynamic light scattering (DLS), nanoparticle tracking analysis (NTA), and transmission electron microscopy (TEM) have been employed to determine the size of NDs [8], [22], [24]. DLS is the most common method used to determine ND size, measuring ensemble changes in scattered light intensity. As the intensity of the scattered light is related to the diameter of the particle to the sixth power [25], this is likely to result in a significant bias towards larger particles. This makes DLS poorly suited for analysing ND samples with a broad size distribution, as is often the case in ND preparations [26]. NTA, on the other hand, is less affected than DLS by samples with a wide range of particle sizes; NTA tracks the Brownian motion of individual particles over time, a distinct advantage over the ensemble measurements taken by DLS. For this study, therefore, NTA was considered to be a more suitable method for measuring ND size due to its ability to track individual particles and thus be less affected by the presence of large particles. It is also able to measure concentration simultaneously [27].

These challenges underscore the potential for significant improvement in the production and characterisation of NDs. This study investigated ND manufacturing process parameters to determine whether repeatability could be improved by controlling multiple parameters. Changes to the size and size distribution of NDs resulting from varying PFC concentration, sonicator tip height, sonication amplitude, total sonication time, and sonication temperature were evaluated. In addition, the power output of a commercial sonicator was measured using calorimetry to better characterise the sonication process and allow for lab-to-lab comparison.

## 2 Materials and methods

### 2.1 Nanodroplet manufacturing apparatus

**Fig. 1:**
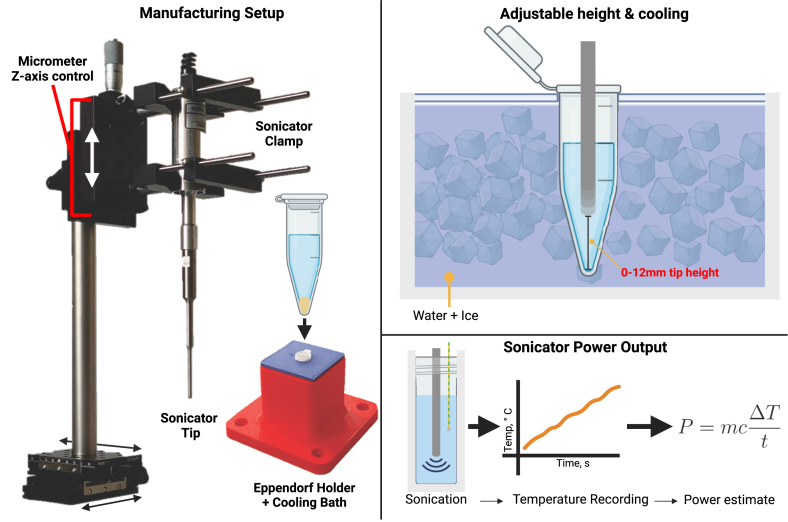
Overview of the manufacturing apparatus. In combination with a custom 3D-printed ice bath with 1.5 mL tube support, the 3-axis control with micrometre Z-axis stage enabled repeatable positioning of the sonicator tip between samples. A thermocouple temperature probe was used to measure changes in temperature over time and subsequently estimate the power output of the sonicator at a given amplitude % setting. Created with BioRender.com.

A manufacturing apparatus was developed to enable repeatable, precise positioning of the sonicator probe (Fig. 1). It utilises a single-axis micrometre translation stage (PT1/M, Thorlabs Inc., USA) in combination with a 3D-printed (Original Prusa i3 MK2, PLA filament) ice bath and a 1.5 mL tube (DNA LoBind®Tubes, Eppendorf SE, Germany) support. This setup ensures that the repeatable positioning and temperature control of the ND manufacturing process are achieved. STL files for both the ice bath and 1.5 mL tube holder lid are available at Mendeley Data (https://dx.doi.org/10.17632/6yh4jzvgz8.3)

### 2.2 Estimating sonicator power output

Sonicators typically have a fixed frequency and maximum amplitude. Power output is adjusted by varying the target tip displacement amplitude, often as a percentage value of the maximum amplitude. For this study, a Model 120 sonic dismembrator (Fisher Scientific, UK) with a 3.2 mm diameter tip was used.

To determine changes in calorimetric energy output during sonication at different amplitude levels (%), the temperature rise over time was measured in a 20 mL volume of room-temperature water within a 30 mL universal container (Starlab (UK) LTD, UK) thermally insulated by styrofoam. A thermocouple data logger (TC-08, Pico Technology, UK) with a type K thermocouple probe (RS Components, UK) was used to record the temperature (°C) every 100 ms. The calorimetric power output in the liquid was used to reflect the power experienced by the solution during nanodroplet production. It is expected to be less than the electrical power input due to energy losses outside of the liquid, such as transducer inefficiencies, acoustic energy losses in the surrounding air or container, and heat losses to the air or container. Sonication intensity was set by specifying a percentage of the maximum displacement amplitude, given as 180 μm by the manufacturer. Sonication intensities of 20, 40, 60, 80 and 100% were tested with a total sonication time of 120 s. A 110 s linear section of the temperature data was selected for each sample and used to calculate the average temperature rise per second. To determine the effect of the composition of the sample on the power output of the sonicator, combinations of water, DSPC:PEG40S, PBS, and PFP were tested (Supplementary Table 1.1). Two sample volumes were used: 0.8 mL to simulate small batch ND manufacturing conditions under pulsed sonication and 5 mL to test large batch ND manufacturing under continuous power output. For the 0.8 mL samples, pulsed sonication of 10 s on, 10 s off at 60% amplitude was used to prevent sample overheating and foam production. 5 mL samples were sonicated at 60% amplitude for 120 s. The average temperature rise per second was calculated from three separate 10 s time windows per sample. The 0.8 mL samples were sonicated in 1.5 mL microcentrifuge tubes. The samples tested are summarised in a table in Supplementary section 1.2. Three independent repeat measurements were made for all samples. All samples were cooled to ∼12.5 °C prior to sonication. To estimate the power output of the sonicator when sonicating different volumes of water, the specific heat of water (4184 J kg^-1^${}^{\circ }$C^-1^) was used throughout [28].

### 2.3 Formulation of lipid-shelled NDs

**Fig. 2:**
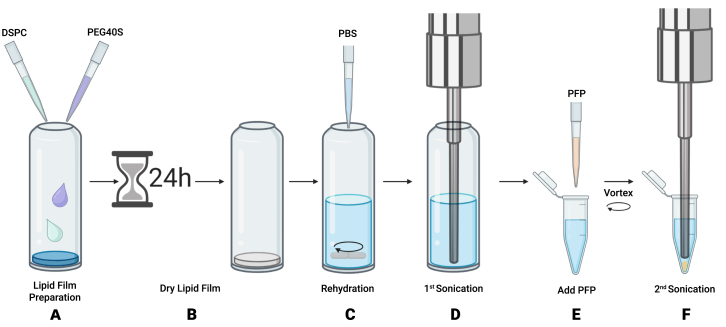
Overview diagram of the process employed for the production of lipid-stabilised perfluorocarbon NDs in a 1.5 mL container. **A.** DSPC and PEG40S are mixed in a 9:1 molar ratio, and **B.** the sample is left to dry overnight. **C.** The dry lipid film is rehydrated with DPBS at 90 °C for 45 min. **D.** A first sonication step is performed to fully disperse the lipids in DPBS. **E.** PFP is added to the lipids in a 1:10 volume ratio in a 1.5 mL tube. The sample is briefly vortexed to form precursor droplets. **F.** A second sonication step forms PFP core, lipid-shelled NDs. Created with BioRender.com.

NDs were manufactured using a method adapted from Ferri et al. [8]. Specifically, 1,2-distearoyl-sn-glycero-3-phosphocholine (DSPC) (Avanti Polar Lipids, USA) and polyoxyethylene (40) stearate (PEG40S) (Sigma-Aldrich, USA) were dissolved in a 7 mL vial (27151, Merck, Germany) in chloroform (Fisher Scientific, UK) at 31.63 mM and 4.89 mM concentration, respectively, to give a 9:1 DSPC:PEG40S molar ratio (621 μL: 447 μL). In previous research, DSPC and PEG40S have been frequently used in a 9:1 molar ratio for the shell of microbubbles (Fig. 2 A) [29]. After mixing, the chloroform was left to evaporate overnight, rendering a dry lipid film (Fig. 2 B). 5 mL of Dulbecco’s phosphate buffered saline without calcium and magnesium (DPBS) (Corning Inc., USA) was used to rehydrate the dry lipid films on a hot plate, stirring at 500 rpm for 45 min at 90 °C to give final concentrations of 3.93 mM and 0.44 mM for DSPC and PEG40S, respectively (Fig. 2 C). To manufacture the ND suspension, a sonicator (Model 120 Sonic Dismembrator, Fisher Scientific, UK) with a 3.2 mm diameter tip (Cat. No. 12911181) was used in two stages. The sonicator has a maximum power input of 120 W, with an operating frequency of 20 kHz. As per the manufacturer’s instruction manual, sonication intensity was controlled by varying the percentage of the maximum probe amplitude (approx. 180 μm). The overall power output was dependent on several factors, including sample viscosity; the instrument automatically varies the power output to maintain the target percentage amplitude. Due to this, the absolute power output for a given amplitude is not defined by the manufacturer.

To fully disperse the lipids in DPBS, the sample was sonicated for 2 min 30 s at room temperature at 40% amplitude setting immediately following the rehydration step (Fig. 2 D). Samples were kept on ice between the first and second sonication stages. Perfluoropentane (dodecafluoropentane or perfluoro-n-pentane, PFP) (STREM Chemicals UK Ltd., UK) was added to the lipid dispersion at volumetric concentrations between 0% v/v and 16.7% v/v and vortexed for 5 s at 500 rpm prior to the second sonication stage (Fig. 2 E). Due to the heat generated by sonication, the sample temperature was controlled using a cooling bath during the second sonication stage. Temperature control is required due to the low boiling point of PFP at 29.2 °C, to prevent or minimise loss of PFP NDs by vaporisation during manufacture. Two pulsed sonication patterns (2 s on, 5 s off and 2 s on, 15 s off) were compared with two types of water bath, i.e. ice and water or ice with saturated saline (∼26% w/v) to assess sample heating of an 80 μL (9.09% v/v) and an 800 μL rehydrated DSPC:PEG40S mixture at 60% amplitude. The 2 s on, 15 s off pulsed sonication pattern combined with a ice-water bath was found to be optimal, keeping samples below 16 °C while preventing sample freezing prior to sonication. Subsequently, the effect of 60% *vs.* 100% amplitude with 2 s on, 15 s off pulse was assessed on the heating of the sample. A type-K thermocouple probe (RS Components, UK) was used to take temperature measurements every 100 ms. A pulsed sonication regime of 2 s on, 15 s off (duty cycle ∼ 13.33%) with a water-ice bath was used in all subsequent ND manufacture experiments. Using 1.5 mL microcentrifuge tubes, NDs were then generated by a second sonication stage (Fig. 2 F).

### 2.4 Effect of varying PFP volumetric concentration and sonicator tip height on ND size characteristics

Volumetric concentrations of PFP of 0, 1.23, 2.44, 4.76, 9.09 and 16.7% v/v were tested and corresponded to 0, 10, 20, 40, 80 and 160 μL of PFP, respectively. The 0% v/v PFP condition was used as a control. As the volumetric concentration of PFP increases, the height of the interface between the PFP precursor droplet phase and bulk lipidic phase also increases. The height of the PFP:water interface compared with the sonicator tip is provided in Supplementary table 1.2. A rehydrated lipid dispersion volume of 800 μL was used for all conditions. Tip heights of 2, 5, 8, 10 and 12 mm were used; a tip height of 0 mm was set at the position where the probe contacted the bottom of the 1.5 mL tube. A total sonication duration of 60 s and a sonication intensity of 60% amplitude were used in these experiments.

### 2.5 Effect of varying sonication duration and sonication intensity on ND size characteristics

Subsequently, the effect of varying total sonication duration (s) and sonication intensity (%) on the size characteristics of NDs was investigated. Both sonication duration and sonication intensity impact the total energy delivered to the system, so they were selected for investigation together. Sonication durations of 15, 30, 45, 60, 75, 90, 105, and 120 s were tested and sonication intensities of 20, 40, 60, 80, and 100% were tested. A PFP volumetric concentration of 4.76% v/v and a sonicator tip height of 12 mm were used for all variables. NDs were kept on ice after manufacture, and three independent repeats were carried out for each condition.

### 2.6 ND characterisation

Nanoparticle tracking analysis (NTA) was performed using a NanoSight NS300 (Malvern Panalytical, Malvern, UK). The NanoSight NS300 was configured with a 488 nm laser and a high-sensitivity sCMOS camera system with a syringe pump. Samples were diluted 1:5000 by volume in DPBS and injected with a NanoSight syringe pump (Malvern Panalytical, Malvern, UK) at pump speed 50 (a.u.) through a low volume flow cell for all measurements. Particles were captured at 30 frames per second, with five 90 s videos per sample. The median hydrodynamic diameter and particle concentration were then calculated using the NTA software. NTA measurements were taken within 15 min of ND production. Due to the upper detection limit of NTA, the presence of particles larger than ∼1 μm diameter was qualitatively assessed using an inverted microscope (Zeiss Axiovert 200). Samples were imaged using a 40× objective (400× magnification) under brightfield illumination.

### 2.7 Statistical analysis

One-way analysis of variance (ANOVA) of NTA samples was performed using GraphPad Prism with a post-hoc Tukey’s multiple comparisons test with p = 0.05 as the significance level.

## 3 Results and discussion

### 3.1 Estimation of sonicator energy output

**Fig. 3:**
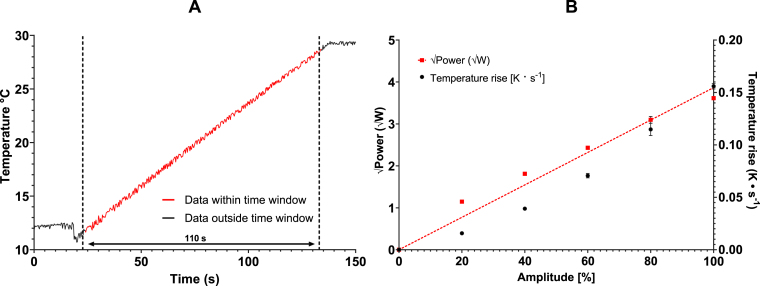
Power output of the sonicator is proportional to the square of the amplitude of tip vibration. **(A)** To understand how power output varied at different sonication intensities, a 20 mL volume of water was sonicated for 120 s. A linear section of the temperature data (110 s) was used to calculate the rate of temperature rise. **(B)** The mean rate of temperature rise (°C s^-1^) changed at different sonication intensities (amplitude, %) in a 20 mL volume of water (n = 3). Amplitudes of 20, 40, 60, 80 and 100% were measured, and assuming the specific heat capacity of water (4184 J⋅kg^-1^⋅K^-1^) a power output (W) was calculated. Assuming power output is proportional to the square of the amplitude, the square of the calculated power output was plotted, resulting in an R^2^ value of 0.95 for $\sqrt{power}$ against sonication amplitude percentage. Error bars are ± 1 SEM.

This study aimed to develop reproducible protocols for obtaining ND emulsions using a commercial sonicator. First, the relationship between its power output and sonication intensity was determined to better characterise the sonicator. A 20 mL volume of room temperature water was sonicated for 120 s between 20% and 100% amplitudes, and the temperature rise was recorded simultaneously. There was a linear increase in temperature with respect to time (R^2^ = 0.9966), indicating a constant power output of the sonicator tip (Fig. 3A). At amplitudes of 20%, 40%, 60%, 80% and 100% the mean rate of temperature increase was 0.016 ± 0.001 °C s^-1^, 0.039 ± 0.001 °C s^-1^, 0.071 ± 0.002 °C s^-1^, 0.115 ± 0.006 °C s^-1^ and 0.156 ± 0.003 °C s^-1^, respectively. Error is ± 1 S.D. of the mean. At 0% amplitude, a temperature change of 0 °C s^-1^ was assumed. Equation (1) was used to calculate the change in heat over time, where P = power in watts (W, J s^-1^), m = mass (kg), c = specific heat (J kg^-1^ °C), ΔT = temperature change (°C), and t = time in seconds (s). Power outputs of 1.42 ± 0.01 W, 1.81 ± 0.02 W, 2.43 ± 0.02 W, 3.10 ± 0.05 W and 3.61 ± 0.02 W were calculated for 20%, 40%, 60%, 80% and 100% amplitudes, respectively. The sonicator tip can be modelled as a piston-like emitter. Pressure by a piston-like emitter has been shown theoretically to be dependent on the amplitude of tip oscillation and hence the square of the amplitude for the power [30]. Thus, the amplitude was plotted against the square of the power, resulting in an R^2^ value of ∼ 0.95 for sonicator amplitude against power^1/2^ (Fig. 3B).

(1) $$\;P=mc\frac{\Delta T}{t}$$

The temperature rise was measured in 5 mL and 0.8 mL sample volumes, corresponding to 30 mL and 1.5 mL sample preparation containers, respectively. The 5 mL volume was chosen to allow continuous sonication; the 0.8 mL volume corresponds to the defined manufacturing volume.

**Fig. 4:**
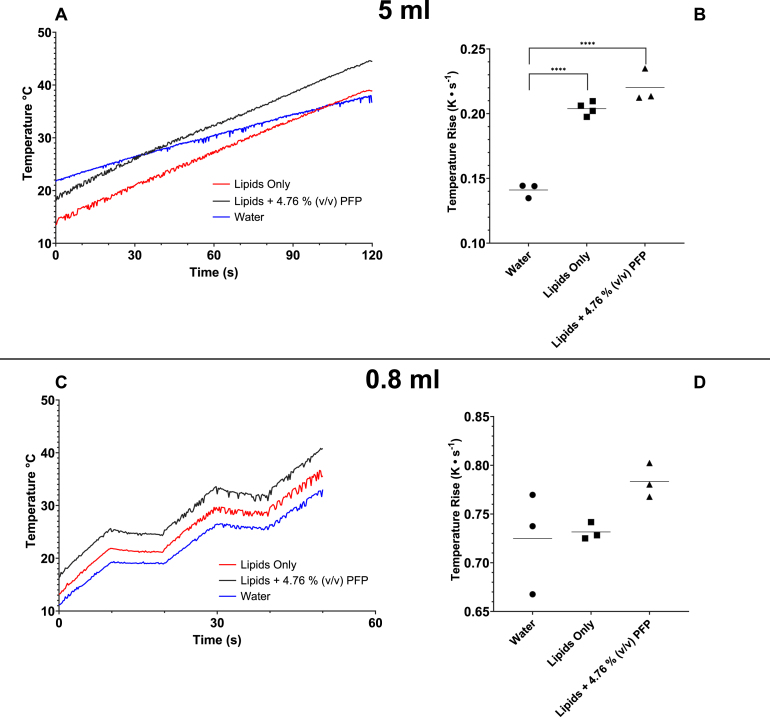
Mean temperature rise during sonication was higher for lipids only and lipids + 4.76% v/v PFP when compared to water. Water, Lipids (rehydrated DSPC:PEG40S 9:1) and Lipids with 4.76% v/v PFP were tested. **(A)** Temperature traces for 5 mL samples sonicated for 120 s at 60% amplitude. **(B)** Mean temperature rise (°C s^-1^) for 5 mL water (0.141 ± 0.005 °C s^-1^, n = 3), 5 mL rehydrated lipids (0.204 ± 0.005 °C s^-1^, n=4) and 5.25 mL rehydrated lipids with 4.76% v/v PFP (0.220 ± 0.01 °C s^-1^, n = 3) sonicated for 120 s at 60% amplitude. Bars show the mean temperature rise for each sample type. A statistically significant difference (p<0.0001) was found between the water and both Lipids-only and Lipids + 4.76% PFP samples. **(C)** Temperature traces for samples sonicated for 60 s with a 10 s on, 10 s off pulse (duty cycle 50%) and 60% amplitude. Pulsed sonication was used to prevent samples from heating too rapidly. **(D)** Mean temperature rise (°C s^-1^) for 0.84 mL water (0.725 ± 0.05 °C s^-1^), 0.8 mL rehydrated lipids (0.730 ± 0.009 °C s^-1^) and 0.8 mL rehydrated lipids with 4.76% v/v PFP (0.783 ± 0.02 °C s^-1^), with n = 3 for all conditions. Bars show the mean temperature rise for each sample type. No statistical difference was found between the sample types. Note that for panel **A** and **C**, a single temperature measurement was selected to represent each sample type.

Volumes of 5 mL water (n = 3), rehydrated lipids in water (n = 4) and rehydrated lipids with 4.76% v/v PFP (n = 3) were sonicated for 120 s at 60% amplitude. (Fig. 4). Similarly to the 20 mL volumes, the increase in temperature during sonication was linear with respect to time for water and lipid/PFP dispersions (Fig. 4A). The mean temperature rise for water, lipids-only, and lipids with 4.76% PFP was 0.141 ± 0.005 °C^-1^, 0.204 ± 0.005 °C s^-1^ and 0.220 ± 0.01 °C s^-1^, respectively (Fig. 4B). A significant difference was found between water *vs.* lipids-only and water *vs.* lipids + 4.76% PFP, with P-values <0.0001. Similarly, 0.8 mL volumes of water, rehydrated lipids and rehydrated lipids with 4.76% v/v PFP at 60% amplitude were tested. The average temperature rise for each sample was calculated from three consecutive 10 s pulses (Fig. 4C). The mean temperature rise for water, lipids-only, and lipids + 4.76% PFP were 0.725 ± 0.05 °C s^-1^, 0.730 ± 0.009 °C s^-1^ and 0.783 ± 0.02 °C s^-1^, respectively (Fig. 4D). No significant differences were found between all sample pairings. The addition of lipids and PFP in the sample increases the sample mass. However, equation (1) predicts that an increase in mass should lead to a reduction in the rate of temperature increase. The increase in temperature change must, therefore, be explained by either a change in the specific heat capacity of the sample or the power output of the sonicator. The presence of lipids and PFP decreases the specific heat capacity of the sample according to the rule of mixtures. This will lead to a more significant temperature increase. However, the mass fraction of water in the sample is large enough to assume that the addition of lipids and PFP does not cause a significant change in heat capacity. Temperature can also affect the specific heat capacity of a solution. However, any changes to specific heat capacity due to temperature changes were assumed to be negligible over the temperature range investigated.

Moreover, the power output of a sonicator tip is affected by the solution viscosity. The amplitude of the vibration is controlled; therefore, more viscous solutions require more input energy to obtain the same displacement of the probe. This results in an increase in power output for a given amplitude. Einstein’s equation for the viscosity of a suspension can be used to estimate the increase in viscosity that the presence of phospholipids and NDs will produce compared with water alone [31]. Previous experimental work on oil-in-water nanoemulsions has shown that the presence of the emulsion increases the viscosity of the solution [32]. This means the lipid and ND solutions require an increased power output from the sonicator compared to water alone, resulting in a larger temperature difference. Additionally, the sonication process is likely to introduce bubbles into the system. These bubbles are likely to last longer in the lipid suspension compared to the water as the bubbles can be stabilised by a lipid monolayer (as is the case in microbubbles and nanobubbles). The presence of bubbles will reduce the water-sonicator coupling efficiency and further increase the required input energy for emulsification.

**Fig. 5:**
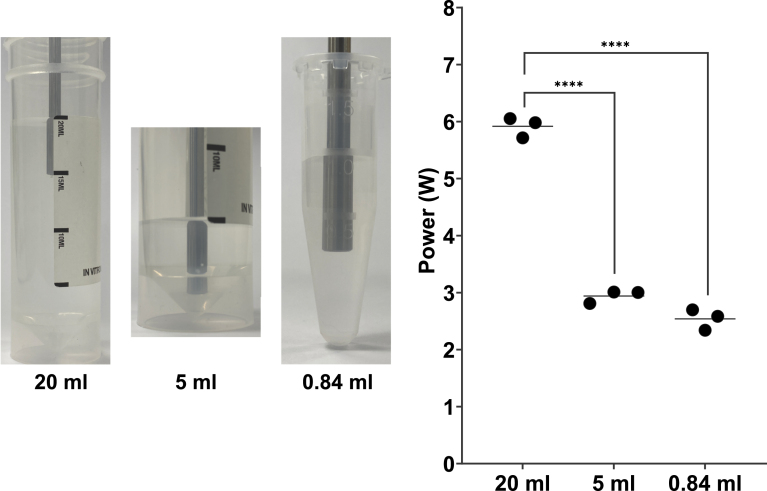
Sonicator power output is reduced for smaller sample volumes at 60% sonication amplitude. **(A)** Images highlighting the containers used to sonicate different water volumes. For the 20 mL and 5 mL samples, a 30 mL universal container was used. A 1.5 mL container was used for the 0.84 mL sample. The sonicator was positioned 12 mm below the water-air interface for the 20 mL and 5 mL samples and 12 mm above the bottom of the 1.5 mL container for the 0.84 mL samples. **(B)** Mean rates of temperature rise (°C s^-1^) and the specific heat capacity of water of 4184 $J\;{\mathrm{kg}}^{-1}\;C{}^{-1}$ were used to calculate a power estimate for each sample type. Bars show the mean power output for each sample type. A statistically significant difference (p < 0.0001) was found between the 20 mL and both 5 mL and 0.84 mL samples.

Differences in the sonicator power output between different volumes of water at 60% amplitude were determined using (1). For the 20 mL and 5 mL samples, the sonicator tip was placed 12 mm below the water-air interface (Fig. 5A). The sonicator tip was placed 12 mm above the base of the 1.5 mL container for the 0.84 mL volume of water (Fig. 5A). Comparing the 20 mL, 5 mL and 0.84 mL volumes of water, the measured calorimetric power output was significantly greater for the 20 mL volume compared to the 5 mL and 0.84 mL volumes (5.92 ± 0.2 W *vs.* 2.94 ± 0.1 W or 2.54 ± 0.2 W, respectively; p<0.0001) (Fig. 5B). This result is as expected, as heat will dissipate more quickly in smaller volumes.

### 3.2 Selection of sonication pulse parameters

**Fig. 6:**
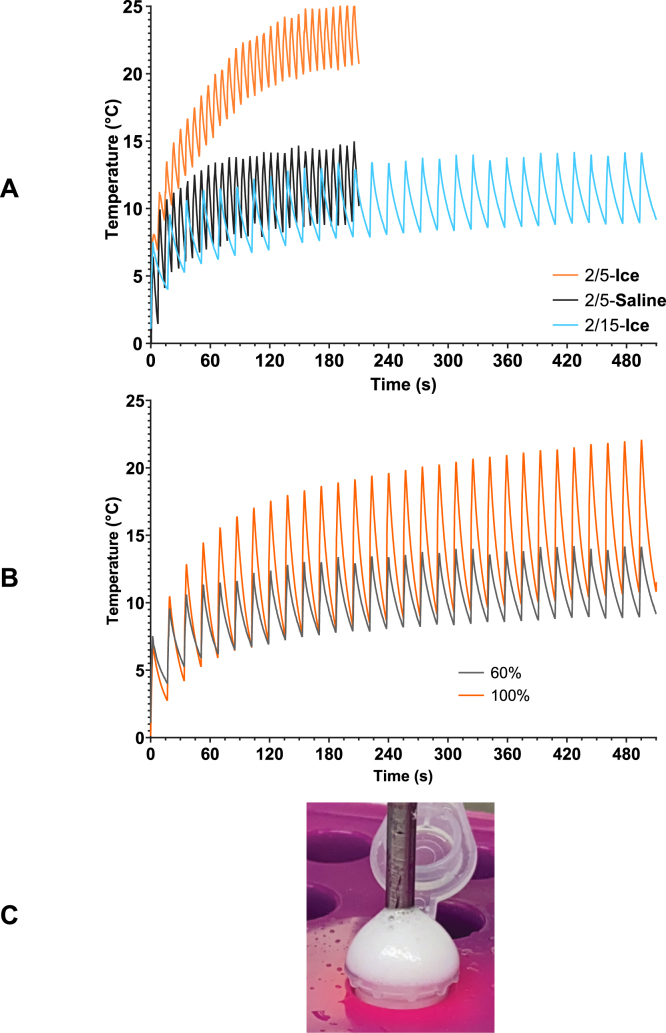
Pulsed sonication of 2 s on, 15 s off with an ice-water bath at 60% sonication amplitude kept temperatures under 16 °C. 800 μL DSPC:PEG40S in DPBS with 9.09% v/v PFP were used. **(A)** Effect of two patterns of pulsed sonication (2 s on, 5 s off *vs.* 2 s on, 15 s off) compared with ice-water (∼ 1 °C) or saturated saline-ice (∼ -8 °C) cooling baths on ND sample temperature. Maximum temperatures for 2/5-ice, 2/5-saline and 2/15-ice were 26.68 °C, 16.04 °C and 15.48 °C, respectively. **(B)** Effect of varying amplitude percentage on the temperature of ND samples. 2 s on, 15 s off pulse parameters were used, and both 60% and 100% amplitudes were tested. Maximum temperatures were 15.48 °C and 25.03 °C for 60% and 100% amplitudes, respectively. All samples were sonicated under identical conditions, with the mean temperature of n = 3 samples plotted. A total sonication on time of 60 s was tested for all samples. **(C)** Image showing the foam production characteristic of sample overheating.

A key limitation of the sonication process is the rise in temperature as a result of the energy output from the sonicator. It is inevitable that any process resulting in large shear forces and cavitation will result in heat production. In the case of our system, which comprises lipids suspended in PBS and PFP, this is a particular problem due to the narrow temperature range in which all components are in their liquid phase. The lipid suspension freezes at just below 0°C; meanwhile, the PFP boils at 29.2°C. Similar to Ferri et al. [8], pulsed sonication was used to mitigate sample heating. Sonication patterns of 2 s on, 5 s off and 2 s on, 15 s off were compared, and two types of water bath containing ice and water or ice with saturated saline (∼26% w/v) were used (Fig. 6A). Upon exposure to the 2 s on and 5 s off pulsed regime in an ice-water bath, the sample temperature was too close to the phase transition temperature of the PFP. As a result, an ice-saline bath was proposed. This was successful in lowering the maximum sample temperature by 10°C; however, on a few occasions, it was observed that the lipid suspension froze before sonication started. An ice-water bath coupled with a longer gap between pulses prevented the lipid suspension’s freezing and allowed the solution to cool between pulses. Therefore, the 2 s on, 15 s off pulsed sonication pattern combined with an ice-water bath (2/15-Ice) was found to be optimal, keeping samples cool while preventing freezing prior to sonication. It is worth noting that temperatures local to the tip of the sonicator are likely to be significantly higher than in the bulk liquid but likely to dissipate quickly.

To confirm if the 100% amplitude tests would overheat the sample, the 2 s on, 15 s off pulsed sonication pattern was tested at 60% and 100% amplitudes (Fig. 6B). Defining a maximum suitable amplitude % was important for the selection of variables in the multiparametric study; if a wide range of amplitude % settings were viable, they could be considered for testing. Sample overheating was marked by substantial foam production during preliminary testing (Fig. 6C). Maximum temperatures of 15.48 °C and 25.03 °C were recorded at 60% and 100% amplitude settings, respectively, with the pulsed sonication regime. These are both within the temperature range acceptable for sonication. However, the 100% amplitude setting generated temperatures much closer to the boiling point of PFP. The 2/15 ice water bath and 60% amplitude setting were selected for all subsequent experiments. In combination, these parameters will reduce the loss of PFP through unnecessary sample heating.

### 3.3 Effect of varying PFP concentration against sonicator tip height on NDs characteristics

NDs were formed by sonication at 60% amplitude with the optimised pulse parameters of 2 s on, 15 s off and a total sonication on time of 60 s.

**Fig. 7:**
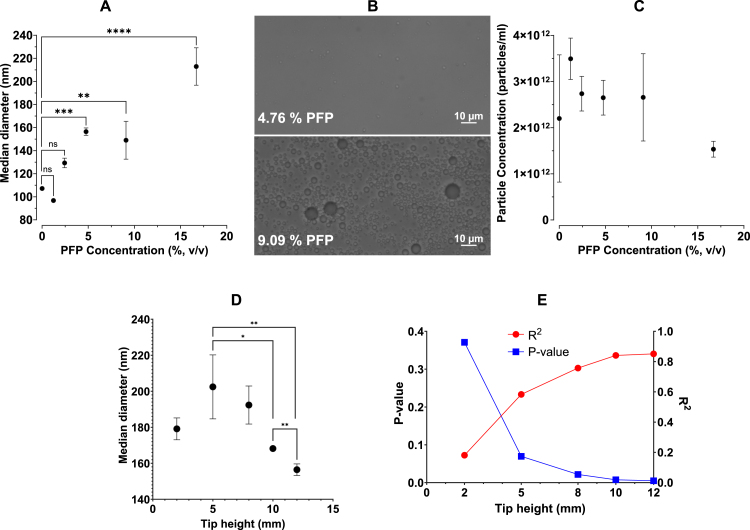
Increasing volumetric concentration of PFP and sonicator tip height significantly affects ND characteristics. n = 3 independent repeats were measured for each pair of tip height and volumetric concentration. **(A)** Effect of varying PFP concentration from 0% to 16.7% v/v on the median ND diameter at 12 mm tip height. **(B)** Microscopy images of ND samples manufactured with a 12 mm tip height and either 4.76% or 9.09% PFP concentration were used in the selection of manufacturing parameters for the sonication intensity *vs.* sonication duration experiment. **(C)** Effect of varying PFP concentration from 0% to 16.7% v/v on mean ND concentration at 12 mm tip height. **(D)** Effect of varying tip height from 2 mm to 12 mm on ND median diameter with 4.76% PFP concentration. Significance values were reported for pairs with P<0.05. All other pairs had a p-value greater than 0.05. There is a weak relationship between variables (R^2^=0.3100) **(E)**. Correlation data between PFP concentration and the median ND diameter at varying tip height from 2 mm to 12 mm. (ns > 0.05, * < 0.05, ** < 0.01, *** < 0.001 and **** < 0.0001) Panels A, C and D show the mean of n = 3 values.

To determine how tip placement affected the formation of NDs, tip heights from 2–12 mm were varied against PFP concentrations from 0% to 16.7% v/v PFP, and the particle diameter and concentration were subsequently measured by NTA (Fig. 7).

Fig. 7A shows that PFP concentration had a very significant effect on the ND diameter. A positive correlation was found between PFP concentration and particle diameter at 12 mm tip height, with a R^2^ value of 0.851. This is consistent with previous results from Ferri et al. [8]. However, there appeared to be a change in behaviour at 4.76% PFP. The characteristics of samples comprising less than 4.76% PFP were found to be not significantly different from the 0% PFP control. As the 0% ND control will consist of lipid-only structures, a lack of a significant difference implies a contribution of lipid-only structures in the samples with less than 4.76% PFP. Lipid-only structures and NDs are indistinguishable using NTA as the technique is limited to size characterisation only and not chemical composition, and so a significant difference in size is required to suggest the presence of NDs. As such, PFP concentrations below 4.76% were rejected as possible parameters for future experiments. These findings are qualitatively consistent with the phase diagram proposed in a study by Gao et al. when interpreting size distributions of polymer-stabilised perfluoropentane NDs. It was suggested that three separate “phases” could be observed depending on the polymer/PFP concentration ratio: a micelle only, ND only and a mixed phase. The existence of each particle was verified using DLS [33].

On examination under a light microscope, a qualitatively significant difference in the number of visible particles between 4.76% and 9.09% PFP was observed. There were significantly more large, circular particles visible in the 9.09% PFP sample compared to the 4.76% PFP sample. (Fig. 7B). This result cannot be observed in (Fig. 7A) as many of these are larger than the 1 μm diameter limit of NTA and therefore would not be detected. There was no significant correlation between PFP concentration and particle concentration (Fig. 7C). The slight decrease in particle concentration as the PFP concentration increases could be attributed to a reduction in the number of particles detected by the NTA due to the increase in particle size.

Whilst PFP concentration had a very clear effect on the ND size, the effect of tip height was more complex to interpret but no less significant. In initial experiments, it appeared visually that there was better mixing of the sample when the tip was positioned higher up in the sample tube. However, using a PFP concentration of 4.76% (v/v), no significant difference in ND diameter with respect to tip height was observed (Fig. 7D, R^2^ = 0.3100). Despite this, at tip heights greater than 5 mm, a decline in median ND diameter was observed. This trend was not observed at 2 mm, where the median ND diameter was smaller than at 5 mm.

**Fig. 8:**
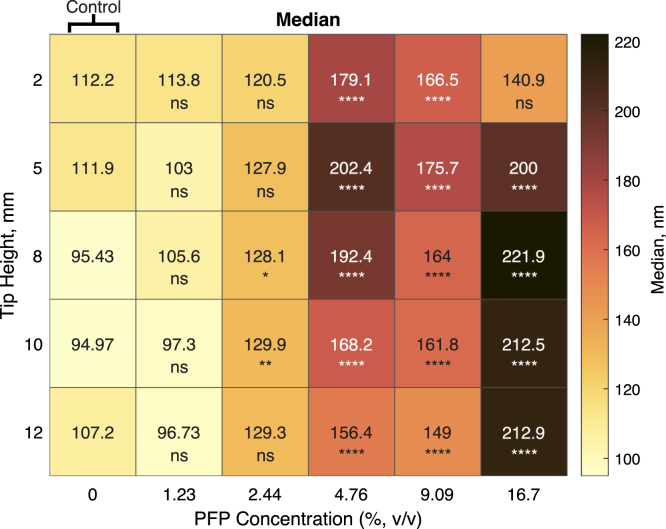
Median ND size increases with increasing PFP volumetric concentration. The sonication duration was 60 s with an amplitude of 60% for all samples. Samples were diluted 1:5000 in DPBS and measured by NTA. Each value is a mean of n = 3 values. Phases did not appear to mix for any sample sonicated with a tip height of 2 mm with 16.7% v/v PFP. For these samples, the upper lipid phase was measured by NTA. Significance is given between each group and the 0% PFP control at the same tip height (ns= not significant, ns > 0.05, * < 0.05, ** < 0.01, *** < 0.001 and **** < 0.0001).

**Fig. 9:**
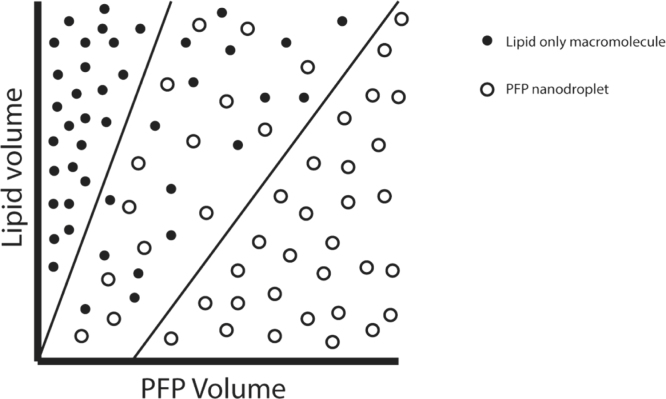
Theoretical diagram to illustrate that as PFP concentration increases, the relative proportion of droplets detected by NTA increases within the range that was examined. At low PFP concentrations, the particle distribution was not significantly different from lipid-only distributions suggesting a significant contribution from lipid-only structures. As PFP concentration increases, the fraction of particles containing PFP (the fraction of PFP-NDs) also increases. Past a certain threshold, the amount of lipid becomes the limiting factor rather than the amount of PFP. The size of the NDs, therefore, changes to accommodate that.

However, the correlation between the PFP concentration and the median ND diameter appears to become stronger as the tip is raised in the Eppendorf tube. For example, the R^2^ value of the relationship between PFP concentration and tip height increased from a value of 0.181 at a 2 mm tip height to a value of 0.851 at a 12 mm tip height (Fig. 7E). The graphs used to generate these values are shown in supplementary Fig. S2. Data reflecting all conditions tested are shown in (Fig. 8). This could be explained by the lipid dispersion above the sonicator tip not mixing adequately with the PFP. This would lead to a larger contribution from lipid-only structures, obscuring any relationship between ND diameter and PFP concentration (Fig. 9). Previous work by Ferdous et al. would seem to support this hypothesis. Modelling mixing in a 1.5 mL Eppendorf tube driven by a sonicator tip showed that the circulation zone (defined as the zone of rotating streamlines with regular flow) within the tube decreases with sonicator tip depth [34]. This suggests that as the sonicator is lowered into the tube, less lipid suspension is subjected to mixing. At 16.7% PFP and 2 mm tip height, there was a clear lipid-PFP phase boundary after sonication. This was the only condition where the sonicator tip was fully submerged in the PFP suggesting a lack of mixing with the lipid suspension above.

### 3.4 Effect of varying sonication duration against intensity on ND characteristics

**Fig. 10:**
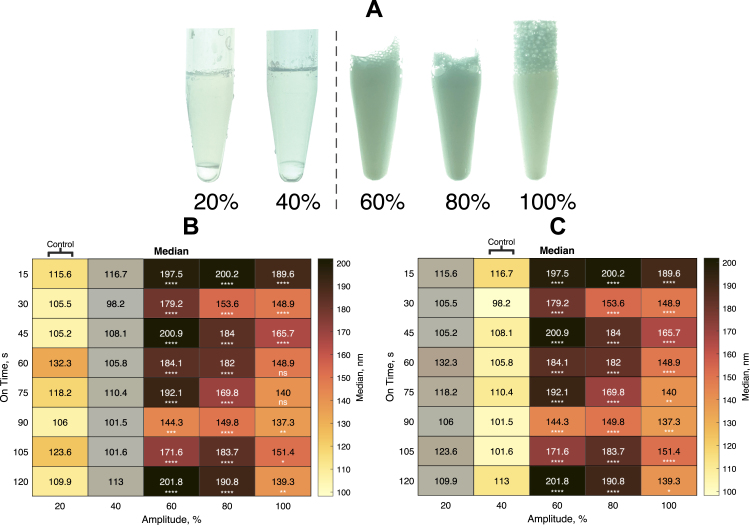
**(A)** Images showing a minimum of 60% sonicator amplitude were required for the PFP and lipidic aqueous phases to mix. For 20% and 40% amplitudes, only the upper lipid phase was analysed. Colourmaps show how the median particle diameter at different sonicator amplitudes (%) and total sonication durations (s) varied. **(B)** Colourmap showing results of a two-way ANOVA with Tukey’s post-hoc test comparing 20% **(B)** and 40% **(C)** amplitude with 60%, 80% and 100% amplitude samples at each time point (ns>0.05, *<0.05, **<0.01, ***<0.001 and ****<0.0001). The tip height was 12 mm with a PFP concentration of 4.76% v/v for all samples. Samples were diluted 1:5000 in DPBS and analysed by NTA. Each colourmap value is a mean of n = 3 values.

The effect of varying sonication duration and intensity on ND size characteristics, using a 4.76% PFP concentration and 12 mm tip height, was subsequently evaluated. Sonication amplitudes of 20% and 40% were insufficient to mix the PFP and lipidic aqueous phases, with 100% amplitude samples showing markedly greater foam production after sonication (Fig. 10A). A minimum amplitude of 60% was required to mix the PFP and lipidic aqueous phases. There was a significant difference in median particle diameter between 20% amplitude and both 60% and 80% at all sonication durations (P<0.001) (Fig. 10B). At 15 s, 30 s, and 45 s sonication duration, there was a significant difference in median particle diameter between 20% and 100% amplitudes (P<0.0001). At 60 s and 75 s sonication duration, there was no significant difference in median particle diameter between samples sonicated at 20% and 100% amplitude (P>0.05). At 90 s, 105 s and 120 s sonication duration, there was a significant difference in the median particle diameter between 20% and 100% amplitude, although the significance was lower (P<0.01 for 90 and 120 s, P<0.05 for 105 s). There was a significant difference in median particle diameter between 40% amplitude and both 60% and 80% at all time points (P<0.0001)(Fig. 10C). At 15 s, 30 s, 45 s, 60 s and 105 s sonication duration, there was a significant difference in median particle diameter between 40% and 100% amplitudes (P<0.0001). At 75 s, 90 s and 120 s sonication duration, there was a significant difference in the median particle diameter between 40% and 100% amplitude, although the significance was lower (P < 0.001 for 90 s, P < 0.01 for 75 s and P < 0.05 for 120 s). Given the fixed 20 kHz frequency, the sonicator amplitude is directly responsible for the cavitation process that results in the shear forces generated within the sample during sonication. It is likely that these shear forces are responsible for the formation of NDs. Both 20% and 40% amplitudes were not sufficient to form NDs, as evidenced by clear stratification between the PFP precursor droplet and lipidic phases that still existed post-sonication. Conversely, 100% amplitude resulted in excessive foaming and so was deemed unsuitable. Overall, 60% amplitude for 90 s was deemed the most appropriate setting for the purpose of in vitro use as the smallest NDs were produced without excessive foaming. Foaming could result in NTA measurement inaccuracies. A sonication duration of 90 s was also chosen because they produced the smallest diameter nanodroplets. Additionally, a sonication duration of 90 s resulted in the lowest standard deviation (±90 nm) at 60% amplitude (Supplementary fig. S3). PFC-NDs with a diameter between 200–350 nm have been shown to passively accumulate in tumour tissue and so this was the target size in this paper [35].

## 4 Limitations

It is important to note that this study is not without its limitations. The main limitation is that this study does not include quantification of the particles above 1 μm, although qualitative analysis was performed. It is also essential to recognise that tip sonication can lead to contamination from probe tip erosion. However, energy dispersive X-ray (EDX) analysis performed on samples prepared using tip sonication showed that titanium contamination contributed to less than 0.1% of the elemental composition. Regular maintenance of sonicator probe tips is essential to minimise degradation of the sonicator tip. In this study, there was a clear relationship between volumetric fraction of PFP used in fabrication of NDs and diameter. Despite this, we did not attempt to measure at an individual particle level the PFP content of individual NDs. Future experiments may confirm this by using, for example, single particle Raman trapping [36], a method that could probe individual ND particles for the presence and concentration of C-F bonds. Additionally, ^19^F NMR can be used to correlate the concentration of PFP within NDs with the NMR peak area of the -$CF_{3}$ group within PFP [20].

## 5 Conclusions

Overall, this work demonstrates that the reproducibility of sonication for ND preparation can be improved and that a reasonable degree of control over nanodroplet size can be achieved. Assessing a range of ND manufacturing parameters allows for a more reproducible combination of parameters to be selected. This is especially important in the medical field, where ND particle size is crucial for determining their vaporisation threshold (the pressure at which the liquid core of the nanodroplets vaporises) and circulation half-life.

This study was conducted with the aim of developing a practical and repeatable manufacturing process for lipid-coated liquid PFP NDs. To achieve this, a 3D-printed tube holder and sonicator positioning system were designed, which not only enabled a reduction in inter-user variability but also increased process reliability. The selection of pulse parameters was shown to be important in maintaining sample temperature during sonication. A 2 s on, 15 s off pulse enabled the sample temperature to be kept under 16 °C at 60% amplitude.

Sufficient ultrasound intensity is needed to mix phases. However, higher-intensity sonication led to increased sample heating and consequent foam production. A 60% amplitude was optimal, ensuring the mixing of phases while minimising sample heating. Calorimetry data showed that increasing sonicator amplitude led to an increase in the rate of temperature rise. The power output from the sonicator was assessed using different volumes of water, lipids, and PFP. As expected, reduced sample volumes led to significant increases in the rate of sample heating, further confirming the importance of sonicator parameter selection.

The selection of optimal manufacturing parameters will depend on the desired application. Smaller nanodroplets are preferable in cases where stability, extravasation potential and the ability to go undetected by the immune system are paramount. However, if used outside of the body, for example, in topical applications requiring ultrasound-responsive particles, a larger size may be preferable to provide a lower ultrasound activation threshold. Increasing PFP concentration was found to increase median nanodroplet diameter and decrease particle concentration. Increasing tip height strengthens this correlation. As such, a tip height of 12 mm was found to be best for repeatable data.

Whilst smaller nanodroplets may be desirable in some applications, nanodroplets created with a PFP concentration below 4.76% v/v particle size were not significantly different from a lipid-only control. Lipid-only controls are rarely reported in the literature. It is crucial that 0% PFP measurements are performed to determine the contribution of non-ND structures contained within these samples, as lipid-only structures are not likely to contribute to the desired therapeutic effect.

## CRediT authorship contribution statement

**Christopher K. Campbell:** Writing – original draft, Visualization, Validation, Methodology, Investigation, Formal analysis, Conceptualization. **Kirsten O’Brien:** Writing – original draft, Visualization, Validation, Methodology, Investigation, Formal analysis, Conceptualization. **Dariusz Kosk:** Methodology, Investigation. **Robin M.H. Rumney:** Writing – review & editing, Supervision. **Peter Glynne-Jones:** Writing – review & editing, Supervision. **Peter R. Birkin:** Writing – review & editing, Conceptualization. **Gareth LuTheryn:** Supervision. **Jeremy S. Webb:** Supervision. **Eleanor Stride:** Writing – review & editing, Supervision. **Dario Carugo:** Writing – review & editing, Supervision. **Nicholas D. Evans:** Writing – review & editing, Supervision, Resources, Project administration, Funding acquisition, Conceptualization.

## Declaration of competing interest

The authors declare the following financial interests/personal relationships which may be considered as potential competing interests: Dario Carugo and Eleanor Stride are inventors on U.S. Provisional Patent No: 63/599,777, which relates to a formulation of ultrasound-responsive nanodroplets. This formulation, however, is not utilised in this paper, and the manufacturing process is not part of the patent. If there are other authors, they declare that they have no known competing financial interests or personal relationships that could have appeared to influence the work reported in this paper.

## Appendix A Supplementary data

MMC S1 Supplementary information.
