# Supplementary material for: Optimising the manufacture of perfluorocarbon nanodroplets through varying sonication parameters
Source: Ultrason Sonochem. 2025 Apr 9;118:107332. doi: 10.1016/j.ultsonch.2025.107332 (PMC12147839; doi:10.1016/j.ultsonch.2025.107332)
Supplement: MMC S1 — Supplementary information. [file mmc1.pdf]

Appendix 1. Supplementary Data

Table 1.1: Summary of samples tested to measure sonicator power output

Sample type	Water	DSPC + PEG40S in DPBS	PFP	Total Volume (mL)
20 mL Water	20	0	0	20
5 mL Water	5	0	0	5
5 mL Lipids	0	5	0	5
5 mL Lipids, 4.76 % (v/v PFP)	0	5	0.25	5.25
0.84 mL Water	0.84	0	0	0.84
0.8 mL Lipids	0	0.8	0	0.8
0.8 mL Lipids, 4.76 % (v/v) PFP	0	0.8	0.04	0.84

Table 1.2: Approximate heights of the PFP-lipid boundary prior to sonication for each PFP concentration investigated.

**PFP concentration,%**	**Approximate height of PFP-lipid boundary,mm**
0	0
1.25	1
2.5	2
5	4
10	6
20	10

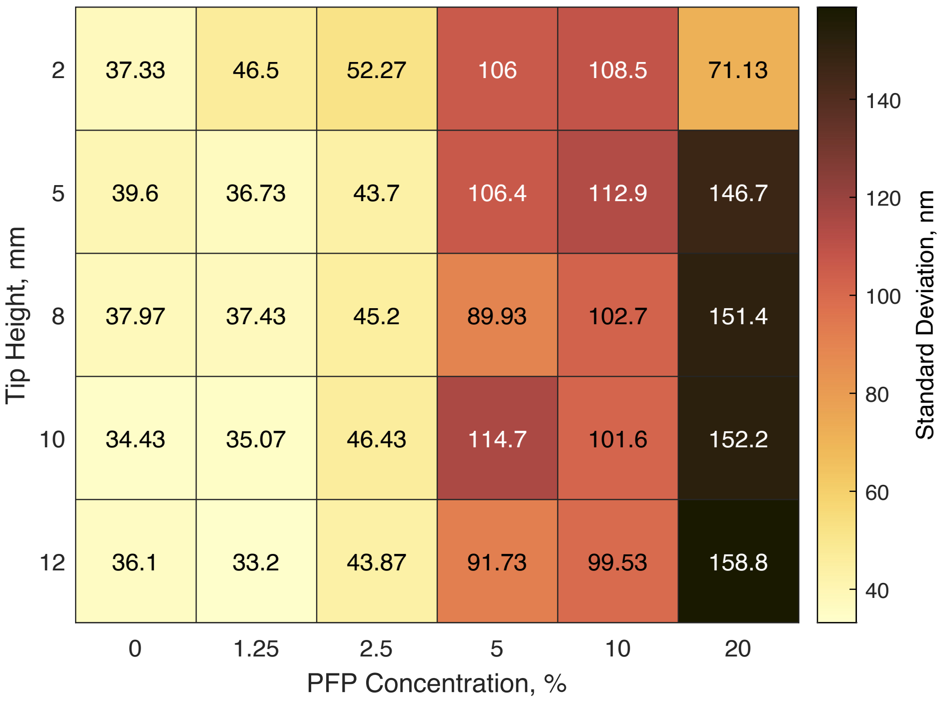

Figure S1: Data for ± 1 standard deviation of the median, n = 3 samples. PFP concentration was varied from 0 % to 16.7 % v/v, and tip height was varied from 2 to 12 mm. The sonication duration was 60 s with an amplitude of 60 % for all samples. Samples were diluted 1:5000 in DPBS and measured by NTA. Each value is a mean of n = 3 values. Phases did not appear to mix for any samples sonicated with a tip height of 2 mm with 16.67 % v/v PFP. For these samples, the upper lipid phase was measured by NTA.

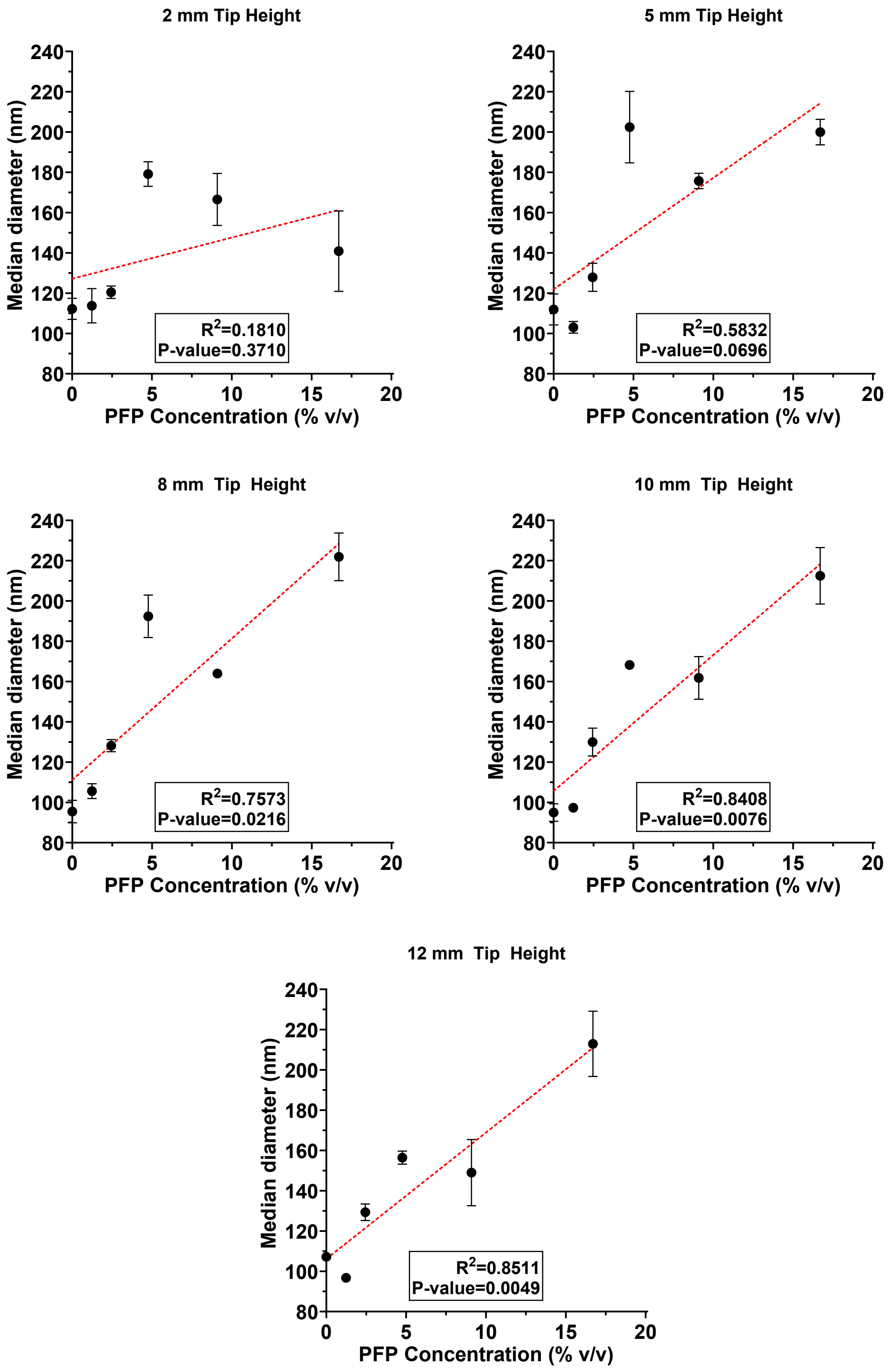

Figure S2: Median diameter at varying PFP concentrations (0 - 16.7% v/v) and varying tip heights (2 - 12 mm). R^2 and P-values are shown for the fitted curve at each tip height.

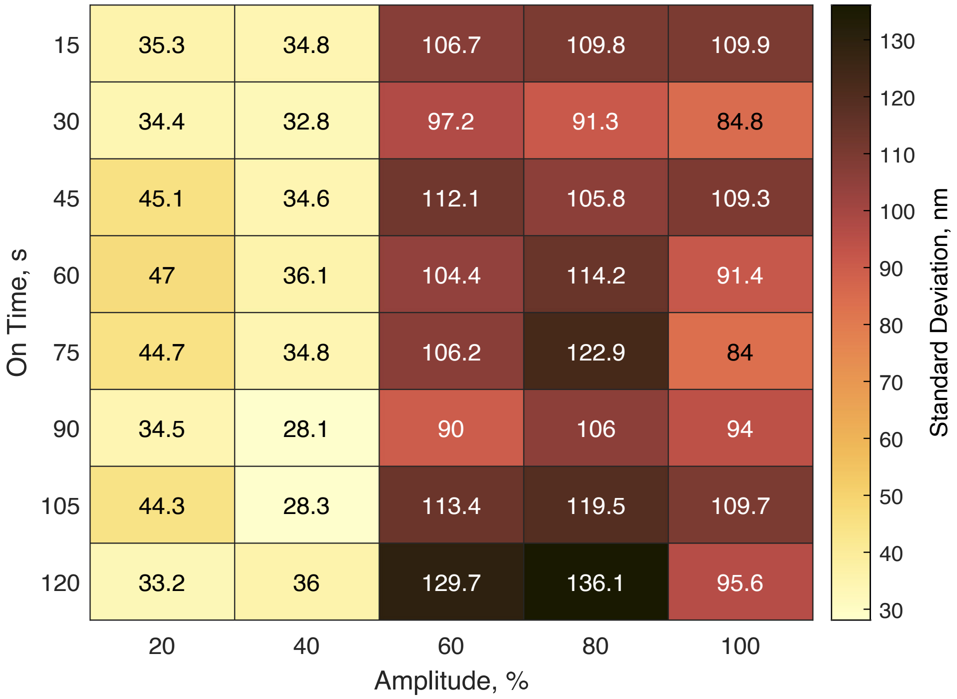

Figure S3: Data for ± 1 standard deviation of the median, n = 3 samples. Sonication duration was varied from 15 s to 120 s, and sonication amplitude was varied from 20 % to 100 %. For all samples, the PFP concentration was 4.76 % v/v, and the tip height was 12 mm. Samples were diluted 1:5000 in DPBS and measured by NTA. Each value is a mean of n = 3 values. Phases did not appear to mix for any samples sonicated with an amplitude of 20 % and 40%. For these samples, the upper lipid phase was measured by NTA.

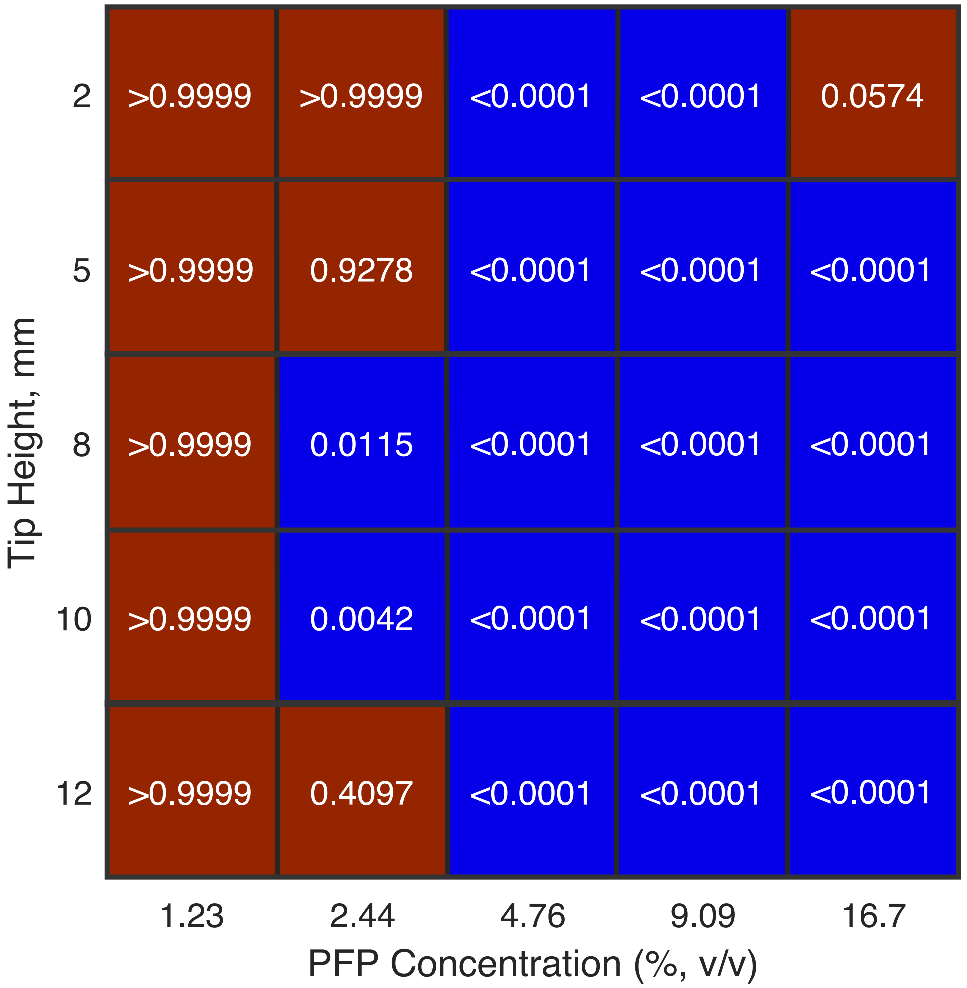

Figure S4: NTA Tip Height *vs.* PFP concentration, comparison to control P-values
